# Supplementary material for: Enhancing combinatorial optimization with classical and quantum generative models
Source: Nat Commun. 2024 Mar 29;15:2761. doi: 10.1038/s41467-024-46959-5 (PMC10980691; doi:10.1038/s41467-024-46959-5)
Supplement: Supplementary file 1 — Supplementary Information [file 41467_2024_46959_MOESM1_ESM.pdf]

Supplementary Information for "Enhancing Combinatorial Optimization with Classical and Quantum Generative Models"

Javier Alcazar,[1,2] Mohammad Ghazi Vakili,[1,3,4] Can B. Kalayci,[1,5] and Alejandro Perdomo-Ortiz[1,*]

[1]*Zapata Computing Canada Inc.,25 Adelaide St E, Suite 1500 Toronto, Ontario M5C 3A1*
[2]*Acadian Asset Management LLC, 24 King William St, London EC4R 9AT, United Kingdom*
[3]*Department of Chemistry, University of Toronto, Toronto, ON, M5G 1Z8, Canada*
[4]*Department of Computer Science, University of Toronto, Toronto, Ontario M5S 2E4, Canada*
[5]*Department of Industrial Engineering, Pamukkale University, Kinikli Campus, 20160, Denizli, Turkey*

Supplementary Note 1. METHODS

A. Generation of portfolio optimization instances

The portfolio optimization problem aims at determining the fractions w_i of a given capital to be invested in each asset i of a universe of N assets, such that the risk $\sigma(\boldsymbol{w})$ for a given level ρ of the expected return $\langle r(\boldsymbol{w})\rangle$ is minimized, constrained to $\sum_i^N w_i = 1$. The problem can be formulated as:

$$\min_{\boldsymbol{w}}\{\sigma^2(\boldsymbol{w}) = \boldsymbol{w}^{\boldsymbol{T}} \cdot \boldsymbol{\Sigma} \cdot \boldsymbol{w} \ : \ \langle r(\boldsymbol{w})\rangle = \boldsymbol{w} \cdot \boldsymbol{r} = \rho\} \tag{1}$$

where the vectors $\boldsymbol{w}$ and $\boldsymbol{r}$ have dimensionality N, $\boldsymbol{\Sigma}$ is the sample covariance matrix obtained from the return time series of pair of asset i and j, and $\boldsymbol{r}$ is the vector of average return of the time series for each asset, with each daily return, r^t, calculated as the relative increment in asset price from its previous day (i.e., $r^t = (p^t - p^{(t-1)})/p^{(t-1)}$, with p^t as the price for a particular asset at time t). The solution to Eq. 1 for a given return level ρ corresponds to the optimal portfolio strategy $\boldsymbol{w}^*$ and the minimal value of this objective function $\sigma(\boldsymbol{w})$ correspond to the portfolio risk and will be denoted by σ^*_ρ.

Note that the optimization task in Eq. 1 has the potential outcome of investing small amounts in a large number of assets as an attempt to reduce the overall risk by "over diversifying" the portfolio. This type of investment strategy can be challenging to implement in practice: portfolios composed of a large number of assets are difficult to manage and may incur in high transaction costs. Therefore, several restrictions are usually imposed on the allocation of capital among assets, as a consequence of market rules and conditions for investment or to reflect investor profiles and preferences. For instance, constraints can be included to control the amount of desired diversification, i.e., modifying bound limits per asset i, denoted by $\{l_i, u_i\}$, to the proportion of capital invested in the investment on individual assets or a group of assets, thus the constraint $l_i < w_i < u_i$ could be considered.

Additionally, a more realistic and common scenario is to include in the optimization task a *cardinality constraint*, which limits directly the number of assets to be transacted to a pre-specified number $\kappa = N$. Therefore, the number of different sets to be treated is $M = \binom{N}{\kappa}$. In this scenario, the problem can be formulated as a Mixed-Integer Quadratic Program (MIQP) with the addition of binary variables $x_i \in \{0, 1\}$ per asset, for $i = 1, ..., N$, which are set to "1" when the i-th asset is included as part of the κ assets, or "0" if it is left out of this selected set. Therefore, valid portfolios would have a number κ of 1's, as specified in the cardinality constraint. For example, for $N = 4$ and $\kappa = 2$, the six different valid configurations can be encoded as $\{0011, 0101, 0110, 1001, 1010, 1100\}$.

The optimization task can then be described as follows

$$\begin{aligned}\min_{\boldsymbol{w},\boldsymbol{x}} \{\sigma^2(\boldsymbol{w}) \quad &: \\ &\langle r(\boldsymbol{w})\rangle = \rho, \\ &l_i x_i < w_i < u_i x_i \quad i = 1, ..., N, \\ &\boldsymbol{1} \cdot \boldsymbol{x} = \kappa\}\,. \end{aligned} \tag{2}$$

In this reformulated problem we denote by $\sigma^*_{\rho,\kappa}$ the minimum portfolio risk outcome from Eq. 2 for a given return level ρ and cardinality κ. The optimal solution vectors $\boldsymbol{w}^*$ and $\boldsymbol{x}^*$ define the portfolio investment strategy. Adding the cardinality constraint

* aperdomo@post.harvard.edu

and the investment bound limits transforms a simple convex optimization problem (Eq. 1) into a much harder non-convex NP-hard problem . For all the problem instance generation in this work we chose $\kappa = N/2$ and the combinatorial nature of the problems lies in the growth of the search space associated with the binary vector $\boldsymbol{x}$, which makes it intractable to exhaustively explore for a number of assets in the few hundreds. The size of the search space here is $M = \binom{N}{N/2}$

It is important to note that given a selection of which assets belong to the portfolio by instantiating $\boldsymbol{x}$ (say with a specific $\boldsymbol{x}^{(i)}$), solving the optimization problem in Eq. 2 to find the respective investment fractions $\boldsymbol{w}^{(i)}$ and risk value $\sigma^{(i)}_{\rho,N/2}$ can be efficiently achieved with conventional quadratic programming (QP) solvers. In this work we used the python module cvxopt [1] for solving this problem. Note that we exploit this fact to break this constrained portfolio optimization problem into a combinatorial intractable one (find best asset selection $\boldsymbol{x}$), which we aim to solve with GEO, and a tractable subroutine which can be solved efficiently with available solvers.

The set of pairwise $(\sigma^{\kappa}_{\rho}, \rho)$, dubbed as the *efficient frontier*, is no longer convex neither continuous in contrast with the solution to problem in Eq. (1).

B. Problem formulation for comparison with state-of-the-art algorithms

To carry out the comparison with State-of-the-Art Algorithms, in line with the formulation used there, we generalizes the problem in Eq. 2 releasing the constraint of a fix level of portfolio return, instead directly incorporating the portfolio return in the objective function, encompassing now two terms: the one on the left corresponding to the portfolio risk as beforeand the one on the right corresponding to the portfolio return. The goal is to balance out both terms such that return is maximized and risk minimized. Lambda is a hyperparameter, named *risk averse*, that controls if an investor wants to give more weight to risk or return. The new formulation reads as follows,

$$\begin{aligned} &\min_{\boldsymbol{w},\boldsymbol{x}} \{\lambda \sigma^2(\boldsymbol{w}) - (1-\lambda)\langle r(\boldsymbol{w})\rangle : \\ &l_i x_i < w_i < u_i x_i \quad i = 1, ..., N, \\ &\qquad\qquad\qquad \mathbf{1} \cdot \boldsymbol{x} = \kappa\} \,. \end{aligned} \tag{3}$$

With the rest of constraints and variables definition as in Supplementary Note 1 A.

1. Performance Metrics

To compare the performance of the proposed GEO with the SOTA metaheuristic algorithms in the literature, the most commonly used performance metrics for the cardinality constrained portfolio optimization problem are used. These metric formulations compute the distance between the heuristic efficient frontier and the unconstrained efficient frontier. Thus, the performance of the algorithms can be evaluated.

Four of these performance metrics (the Mean, Median, Minimum and Maximum in Table 1) are based on the so-called Performance Deviation Errors (PDE). These PDE metrics were formulated by Chang [2] as follows:

$$\mathrm{PDE_i} = \min\left(\left| \frac{100\,(\mathrm{x_i} - \mathrm{x_i^*})}{\mathrm{x_i^*}} \right|, \left| \frac{100\,(\mathrm{y_i} - \mathrm{y_i^*})}{\mathrm{y_i^*}} \right| \right) \tag{4}$$

$$\begin{aligned} x_i^* &= X_{k_y} + \frac{\left(X_{j_y} - X_{k_y}\right)\left(y_i - Y_{k_y}\right)}{\left(Y_{j_y} - Y_{k_y}\right)} \\ y_i^* &= Y_{k_x} + \frac{\left(Y_{j_x} - Y_{k_x}\right)\left(x_i - X_{k_x}\right)}{\left(X_{j_x} - X_{k_x}\right)} \\ j_y &= {}_{l=1,\ldots,\varepsilon^* \wedge Y_l \geq y_i} Y_l \\ k_y &= {}_{l=1,\ldots,\varepsilon^* \wedge Y_l \leq y_i} Y_l \\ j_x &= {}_{l=1,\ldots,\varepsilon^* \wedge X_l \geq x_i} X_l \\ k_x &= {}_{l=1,\ldots,\varepsilon^* \wedge X_l \leq x_i} X_l \end{aligned} \tag{5}$$

where the pair $(X_l, Y_l)(l = 1, ..., \varepsilon^*)$ represents the point on the standard efficient frontier and the pair $(x_i, y_i)(i = 1, ..., \varepsilon)$ represents the point on the heuristic efficient frontier. Here, ε^* denotes the number of points on the standard efficient frontier

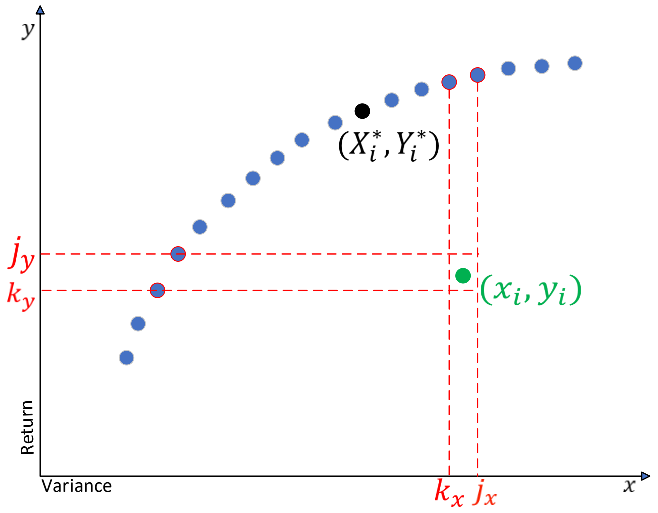

Supplementary Figure 1. A graphical demonstration of indices used for performance metrics calculation

while ε denotes the number of points on the heuristic efficient frontier. The mean, median, minimum, and maximum of the PDE can be used to compare the performance of the algorithms.

Later, three additional performance measures (MEUCD: Mean Euclidean Distance, VRE: Variance of Return Error, MRE: Mean Return Error) were formulated by Cura [3] as follows:

$$\text{MEUCD} = \frac{\sum_{i=1}^{\varepsilon} \sqrt{(X_i^* - x_i) + (Y_i^* - y_i)}}{\varepsilon} \tag{6}$$

$$\text{VRE} = \frac{\sum_{i=1}^{\varepsilon} 100|X_i^* - x_i|/x_i}{\varepsilon} \tag{7}$$

$$\text{MRE} = \frac{\sum_{i=1}^{\varepsilon} 100|Y_i^* - y_i|/y_i}{\varepsilon} \tag{8}$$

where (X_i^*, Y_i^*) is the standard point closest to the heuristic point (x_i, y_i). Figure 1 shows a graphical representation of the indices used to calculate the performance metrics for the convenience of the reader and the values for TN-GEO and all the other SOTA optimizers are reported in Table 1.

C. Quantum-Inspired Generative Model in TN-GEO

The addition of a probabilistic component is inspired by the success of Bayesian Optimization (BO) techniques, which are among the most efficient solvers when the performance metric aims to find the lowest minimum possible within the least number of objective function evaluations. For example, within the family of BO solvers, GPyOpt [4] uses a Gaussian Process (GP) framework consisting of multivariate Gaussian distributions. This probabilistic framework aims to capture relationships among the previously observed data points (e.g., through tailored kernels), and it guides the decision of where to sample the next evaluation with the help of the so called acquisition function. GPyOpt is one of the solvers we use to benchmark the new quantum-enhanced strategies proposed here.

Although the GP framework in BO techniques is not a generative model, we explore here the powerful unsupervised machine learning framework of generative modeling in order to capture correlations from an initial set of observations and evaluations of the objective function (step 1-4 in Fig. 1).

For the implementation of the quantum-inspired generative model at the core of TN-GEO we follow the procedure proposed and implemented in Ref. [5]. Inspired by the probabilistic interpretation of quantum physics via Born's rule, it was proposed that one can use the Born probabilities $|\Psi(\boldsymbol{x})|^2$ over the 2^N states of an N qubit system to represent classical target probability distributions which would be obtained otherwise with generative machine learning models. Hence,

$$P(\boldsymbol{x}) = \frac{|\Psi(\boldsymbol{x})|^2}{Z}, \text{ with } Z = \sum_{\boldsymbol{x} \in \mathcal{S}} |\Psi(\boldsymbol{x})|^2, \tag{9}$$

with $\Psi(\boldsymbol{x}) = \langle \boldsymbol{x}|\Psi\rangle$ and $\boldsymbol{x} \in \{0,1\}^{\otimes N}$ are in one-to-one correspondence with decision variables over the investment universe with N assets in our combinatorial problem of interest here. In Ref. [5] these quantum-inspired generative models were named as *Born machines*, but we will refer to them hereafter as *tensor-network Born machines* (TNBM) to differentiate it from the *quantum circuit Born machines* (QCBM) proposal [6] which was developed independently to achieve the same purpose but by leveraging quantum wave functions from quantum circuits in NISQ devices. As explained in the main text, either quantum generative model can be adapted for the purpose of our GEO algorithm.

On the grounds of computational efficiency and scalability towards problem instances with large number of variables (in the order of hundreds or more), following Ref. [5] we implemented the quantum-inspired generative model based on Matrix Product States (MPS) to learn the target distributions $|\Psi(\boldsymbol{x})|^2$.

MPS is a type of TN where the tensors are arranged in a one-dimensional geometry. Despise its simple structure, MPS can efficiently represent a large number of quantum states of interest extremely well [7]. Learning with the MPS is achieved by adjusting its parameters such that the distribution obtained via Born's rule is as close as possible to the data distribution. MPS enjoys a direct sampling method that is more efficient than other Machine Learning techniques, for instance, Boltzmann machines, which require Markov chain Monte Carlo (MCMC) process for data generation.

The key idea of the method to train the MPS, following the algorithm on paper [5], consists of adjusting the value of the tensors composing the MPS as well as the bond dimension among them, via the minimization of the negative log-likelihood function defined over the training dataset sampled from the target distribution. For more details on the implementation see Ref. [5] and for the respective code see Ref. [8].

D. NADE-GEO - A Binary Generative Model for GEO Optimizer

This section provides a detailed overview of the NADE-GEO model, a binary generative model used for GEO optimization. We discuss the formulation, methodology, and motivation behind the model.

Neural Autoregressive Distribution Estimator (NADE) is a generative model introduced by Uria et al. [9]. The fundamental idea behind NADE is to model the distribution of high-dimensional data (such as images) by decomposing the joint distribution into a product of conditionals, each of which is modeled by a neural network. This process is referred to as autoregression. For a D-dimensional binary vector x, the joint distribution can be factorized as:

$$P(x) = \prod_{d=1}^{D} P(x_d|x_1, ..., x_{d-1}). \tag{10}$$

NADE uses a shared neural network whose hidden layer is masked to ensure that only the relevant inputs $(x_1, ..., x_{d-1})$ are used to compute each conditional probability. This results in a more efficient and scalable model.

A NADE model is trained like any other neural network using stochastic gradient descent (SGD). The objective is to maximize the training data's log-likelihood, corresponding to minimizing the binary cross-entropy between the data and the model's predictions.

Sampling in generative models like NADE is the process of creating new data instances that resemble the training data based on the learned distribution. After the model has been trained and the joint distribution of the data has been estimated, we can generate new samples from this distribution.

In the case of NADE, due to the autoregressive nature of the model, sampling is a sequential process. We begin by sampling the first element of the vector x, x_1, from its marginal distribution, $P(x_1)$. Then, we proceed by sampling each subsequent element x_d from the corresponding conditional distribution, $P(x_d|x_1, ..., x_{d-1})$, where the conditionals are given by the previously sampled elements. This process is repeated until a sample for every element of the vector has been drawn.

The strength of NADE lies in the fact that the ordering of the dimensions (or features) can have a substantial impact on the model's performance. The choice of ordering can be problem-dependent, and exploiting this aspect can potentially yield more accurate samples and better model performance.

E. Classical Optimizers

1. GPyOpt Solver

GPyOpt [4] is a Python open-source library for Bayesian Optimization based on GPy and a Python framework for Gaussian process modelling. For the comparison exercise in TN-GEO as a stand-alone solver here are the hyperparameters we used for the GPyOpt solver:

- Domain: to deal with the exponential growth in dimensionality, the variable space for n number of assets was partitioned as the cartesian product of n 1-dimensional spaces.
- Constraints: we added two inequalities in the number of assets in a portfolio solution to represent the cardinality condition.
- Number of initial data points: 10
- Acquisition function: Expected Improvement

2. Simulated Annealing Solver

For simulated annealing (SA) we implemented a modified version from Ref. [10]. The main change consists of adapting the update rule such that new candidates are within the valid search space with fixed cardinality. The conventional update rule of single bit flips will change the Hamming weight of $\boldsymbol{x}$ which translates in a portfolio with different cardinality. The hyperparameters used are the following:

- Max temperature in thermalization: 1.0
- Min temperature in thermalization: 1e-4

3. Conditioned Random Solver

This solver corresponds to the simplest and most naive approach, while still using the cardinality information of the problem. In the *conditioned random solver*, we generate, by construction, bitstrings which satisfy the cardinality constraint. Given the desired cardinality $\kappa = N/2$ used here, one starts from the bitstring with all zeros, $\boldsymbol{x}_0 = 0 \cdots 0$, and flips only $N/2$ bits at random from positions containing 0's, resulting in a valid portfolio candidate $\boldsymbol{x}$ with cardinality $N/2$.

4. Random Solver

This solver corresponds to the simplest approach without even using the cardinality information of the problem. In the *random solver*, we generate, by construction, bitstrings randomly selected from the 2^N bitstrings of all possible portfolios, where N is the number of assets in our investment universe.

F. Algorithm Methodology for TN-GEO as a booster

As explained in the main text, in this case it is assumed that the cost of evaluating the objective function is not the major computational bottleneck, and consequently there is no practical limitations in the number of observations to be considered.

Following the algorithmic scheme in Fig. 1, we describe next the details for each of the steps in our comparison benchmarks:

0 *Build the seed data set,* $\{\boldsymbol{x}^{(i)}\}_{\text{seed}}$ *and* $\{\sigma^{(i)}_{\rho,N/2}\}_{\text{seed}}$. For each problem instance defined by ρ and a random subset with N assets from the S&P 500, gather all initial available data obtained from previous optimization attempts with classical solver(s). In our case, for each problem instances we collected 10,000 observations from the SA solver. These 10,000 observations corresponding to portfolio candidates $\{\boldsymbol{x}^{(i)}\}_{\text{init}}$ and their respective risk evaluations $\{\sigma^{(i)}_{\rho,N/2}\}_{\text{init}}$ were sorted and only the first $n_{\text{seed}} = 1,000$ portfolio candidates with the lowest risks were selected as the seed data set. This seed data set is the one labeled as $\{\boldsymbol{x}^{(i)}\}_{\text{seed}}$ and $\{\sigma^{(i)}_{\rho,N/2}\}_{\text{seed}}$ in the main text and hereafter. The idea of selecting a percentile of the original data is to provide the generative model inside GEO with samples which are the target samples to be generated. This percentile is a hyperparameter and we set it 10% of the initial data for our purposes.

1 *Construct of the softmax surrogate distribution:* Using the seed data from step 0, we construct a softmax multinomial distribution with n_{seed} classes - one for each point on the seed data set. The probabilities outcome associated with each of these classes in the multinomial is calculated as a Boltzmann weight, $p_i = \frac{e^{-\overline{\sigma}^{(i)}_{\rho,\kappa}}}{\sum_{j=1}^{n_{\text{seed}}} e^{-\overline{\sigma}^{(j)}_{\rho,\kappa}}}$. Here, $\overline{\sigma}^{(i)}_{\rho,\kappa} = \sigma_{\rho,\kappa}(\boldsymbol{x}^{(i)})/T$, and T is a "temperature" hyperparameter. In our simulations, T was computed as the standard deviation of the risk values of this seed data set. In Bayesian optimization methods the surrogate function tracks the landscape associated with the values of the objective function (risk values here). This *softmax surrogate* constructed here by design as a multinomial distribution from the seed data observations serves the purpose of representing the objective function landscape but in probability space. That is, it will assign higher probability to portfolio candidates with lower risk values. Since we will use this softmax surrogate to generate the training data set, this bias imprints a preference in the quantum-inspired generative model to favor low-cost configurations.

2 *Sample from softmax surrogate*. We will refer to these samples as the training set since these will be used to train the MPS-based generative model. For our experiments here we used $n_{\text{train}} = 10000$ samples.

3 *Use the n_{train} samples* from the previous step to train the MPS generative model.

4 *Obtain n_{MPS} samples* from the generative model which correspond to the new list of potential portfolio candidates. In our experiments, $n_{\text{MPS}} = 4000$. For the case of 500 assets, as sampling takes sensibly longer because of the problem dimension, this value was reduced to 400 to match the time in SA.

5 *Select new candidates:* From the n_{MPS} samples, select only those who fulfill the cardinality condition, and which have not been evaluated. These new portfolio candidates $\{\boldsymbol{x}^{(i)}\}_{\text{new}}$ are saved for evaluation in the next step.

6 *Obtain risk value for new selected samples:* Solve Eq. 2 to evaluate the objective function (portfolio risks) for each of the new candidates $\{\boldsymbol{x}^{(i)}\}_{\text{new}}$. We will denote refer to the new cost function values by $\{\sigma^{(i)}_{\rho,N/2}\}_{\text{new}}$.

7 *Merge the new portfolios*, $\{\boldsymbol{x}^{(i)}\}_{\text{new}}$, and their respective cost function evaluations, $\{\sigma^{(i)}_{\rho,N/2}\}_{\text{new}}$ with the seed portfolios, $\{\boldsymbol{x}^{(i)}\}_{\text{seed}}$, and their respective cost values, $\{\sigma^{(i)}_{\rho,N/2}\}_{\text{seed}}$, from step 0 above. This combined super set is the *new initial data set*.

8 *Use the new initial data set from step 7* to start the algorithm from step 1. If a desired minimum is already found or if no more computational resources are available, one can decide to terminate the algorithm here. In all of our benchmark results reported here when using TN-GEO as a booster from SA intermediate results, we only run the algorithm for this first cycle and the minima reported for the TN-GEO strategy is the lowest minimum obtained up to step 7 above.

G. Algorithm Methodology for TN-GEO as a stand-alone solver

This section presents the algorithm for the TN-GEO scheme as a stand-alone solver. In optimization problems where the objective function is inexpensive to evaluate, we can easily probe it at many points in the search for a minimum. However, if the cost function evaluation is expensive, e.g., tuning hyperparameters of a deep neural network, then it is important to minimize the number of evaluations drawn. This is the domain where optimization technique with a Bayesian flavour, where the search is being conducted based on new information gathered, are most useful, in the attempt to find the global optimum in a minimum number of steps.

The algorithmic steps for TN-GEO as a stand-alone solver follows the same logic as that of the solver as a booster described Sec. Supplementary Note 1 F. The main differences between the two algorithms rely on step 0 during the construction of the *initial data set* and *seed data set* in step 0, the temperature use in the softmax surrogate in step 1, and a more stringent selection criteria in step 5. Since the other steps remain the same, we focus here to discuss the main changes to the algorithmic details provided in Sec. Supplementary Note 1 F.

0 *Build the seed data set:* since evaluating the objective function could be the major bottleneck (assumed to be expensive) then we cannot rely on cost function evaluations to generate the seed data set. The strategy we adopted is to initialize the algorithm with samples of bitstrings which satisfy the hard constraints of the problem. In our specific example, we can easily generate n_{seed} random samples, $\mathcal{D}_0 = \{\boldsymbol{x}^{(i)}\}_{\text{seed}}$, which satisfy the cardinality constraint. Since all the elements in this data set hold the cardinality condition, then maximum length n_{seed} of $\mathcal{D}_0$ is $\binom{N}{\kappa}$. In our experiments, we set the number of samples $n_{\text{init}} = 2,000$, for all problems considered here up to $N = 100$ assets

1 *Construct the softmax surrogate distribution:* start by constructing a uniform multinomial probability distribution where each sample in $\mathcal{D}_0$ has the same probability. Therefore, for each point in the seed data set its probability is set to $p_0 = 1/n_{\text{seed}}$. As in TN-GEO as a booster, we will attempt to generate a softmax-like surrogate which favors samples with low cost value, but we will slowly build that information as new samples are evaluated. In this first iteration of the algorithm, we start by randomly selecting a point $\boldsymbol{x}^{(1)}$ from $\mathcal{D}_0$, and we evaluate the value of its objective function $\sigma^{(1)}$ (its risk value in our specific finance example). To make this point $\boldsymbol{x}^{(1)}$ stand out from the other unevaluated samples, we set its probability to be twice that of any of the remaining $n_{\text{seed}} - 1$ points in $\mathcal{D}_0$. Since we increase the probability of one of the points, we need to adjust the probability of the $n_{\text{seed}} - 1$ from p_0 to $p_0^{'}$, and if we assume the probability weights for observing each point follows a multinomial distribution with Boltzmann weights, under these assumptions, and making by fixing the temperature hyperparameter we can solve for the reference "risk" value $\sigma^{(0)}$ associated to all the other $n_{\text{seed}} - 1$ points as shown below. It is important to note that $\sigma^{(0)}$ is an artificial reference value which is calculated analytically and does not require a call to the objective function (in contrast to $\sigma^{(1)}$). Here, $\mathcal{N}$ is the normalization factor of the multinomial and T is the temperature hyperparameter which, as in the case of TN-GEO as a booster, can be adjusted later in the algorithm as more data is seen. Due to the lack of initial cost function values, in order to set a relevant typical "energy" scale in this problem, we follow the procedure in Ref. [11] where it is set to be the square root of the mean of the covariance matrix defined in Eq. 1, as this matrix encapsulates the risk information (volatility) as stated in the Markowitz's model.

$$
\begin{aligned}
&\begin{cases} (n_{\text{seed}} - 1)p_0^{'} + p_1 = 1 \\ p_1 = 2 \cdot p_0^{'} \end{cases} \Rightarrow \begin{cases} p_0^{'} = 1/(1 + n_{\text{seed}}) \\ p_1 = 2/(1 + n_{\text{seed}}) \end{cases} \\
&\begin{cases} \mathcal{N} = (n_{\text{seed}} - 1)e^{-\sigma^{(0)}/T} + e^{-\sigma^{(1)}/T} \\ p_1 = e^{-\sigma^{(1)}/T}/\mathcal{N} \\ p_0^{'} = e^{-\sigma^{(0)}/T}/\mathcal{N} \end{cases} \Rightarrow \\
&\begin{cases} \mathcal{N} = (n_{\text{seed}} + 1) \cdot e^{-\sigma^{(1)}/T}/2 \\ \sigma^{(0)} = T \cdot \log 2 + \sigma^{(1)} \end{cases}
\end{aligned}
\tag{11}
$$

2 *Generate training set:* same as in TN-GEO as a booster (see Supplementary Note 1 F).

3 *Train MPS:* same as in TN-GEO as a booster (see Supplementary Note 1 F).

4 *Generate samples from trained MPS:* same as in TN-GEO as a booster (see Supplementary Note 1 F).

5 *Select new candidates from trained MPS:* In contrast to TN-GEO as a booster we cannot afford to evaluate all new candidates coming from the MPS samples. In our procedure we selected only two new candidates which must meet the cardinality constraint. For our procedure these two candidates correspond to the most frequent sample ("exploitation") and the least frequent sample ("exploration"). If all new samples appeared with the same frequency, then we can select two samples at random. In the case where no new samples were generated, we choose them from the unevaluated samples of the original seed data set in $\mathcal{D}_0$

6 *Obtain risk value for new selected samples:* same as in TN-GEO as a booster (see Supplementary Note 1 F).

7 *Merge the new portfolios with seed data set from step 0* same as in TN-GEO as a booster (see Supplementary Note 1 F).

8 *Restart next cycle of the algorithm with the merge data set as the new seed data set:* same as in TN-GEO as a booster (see Supplementary Note 1 F).

H. Hyperparameter Tuning Details

In this Supplementary Information, we provide additional details on the hyperparameter tuning process for TN-GEO and NADE-GEO. We used Taguchi optimization for TN-GEO, fine-tuning the hyperparameters related to the model's trainability and sampling performances. Additionally, Optuna was employed for hyperparameter tuning in NADE-GEO, optimizing the relevant parameters for the model's architecture and trainability.

1. TN-GEO

In our study on TN-GEO, we utilized Taguchi optimization to fine-tune the hyperparameters. Specifically, we focused on optimizing the number of initial samples, the number of samples outside of the training set, the number of resampled candidates, the total number of candidates, the number of resample parameters, and the additive resample value parameter. By adjusting these parameters using Taguchi's five levels, we effectively optimized TN-GEO's performance and adaptability to different data scenarios. Overall, Taguchi optimization enabled us to systematically explore and optimize these key parameters, resulting in improved performance for TN-GEO.

2. NADE-GEO

In addition to using Taguchi optimization for TN-GEO, we employed Optuna for hyperparameter tuning in NADE-GEO. Specifically, we focused on optimizing the number of layers, learning rate, and number of selector parameters in GEO. These hyperparameters play a crucial role in shaping the architecture and training dynamics of NADE-GEO. By leveraging Optuna, we systematically explored different combinations of these hyperparameters, enabling us to enhance the performance and accuracy of NADE-GEO. The integration of Optuna with NADE-GEO allowed for efficient and effective hyperparameter tuning, leading to improved results in our study.

Supplementary Note 2. RELATIVE TN-GEO ENHANCEMENT

Figure 2 represents the relative performance within the strategies 1 and 2 referred to in section II A.

Supplementary Note 3. ALL ALGORITHMS COMPARISON

In section II C we presented all the nine different leading SOTA optimizers plus NADE-GEO covering a broad spectrum of algorithmic strategies for this specific combinatorial problem. To be explicit about the algorithms involved in this section, we present the list and references of those algorithms once again: GTS [2]; IPSO [12]; IPSO-SA [13]; PBILD [14]; GRASP [15]; ABCFEIT [16]; AAG [17]; VNSQP [18]; ABC-HP [19]; and NADE-GEO corresponding to GEO when used in conjunction with the classical generative model NADE proposed in Ref. [9].

The test data and the comparison metrics used are the same as the ones presented in section II C. Furthermore, the same tables format and convention regarding colors are respected here.

Supplementary Tables 1 and 2 show the comparison between all SOTA algorithms, GEO-NADE and TN-GEO, whereas Supplementary Table 3 shows the comparison between all the algorithms and NADE-GEO.

From all the entries in this table, 69% of them correspond to italic entries, where TN-GEO either wins or draws, which is a significant percentage giving that these optimizers are among the best reported in the last decades.

In Supplementary Table 2, we show a pairwise comparison of TN-GEO against each of the SOTA optimizers. As before, this table reports the number of times TN-GEO wins, loses, or draws compared to results reported for the other optimizers across all the performance metrics and for all five different market indexes. Note that since not all the performance metrics are reported for all the solvers and market indexes, the total number of wins, draws, or losses varies. Therefore, we report the overall percentage of wins plus draws in the same table in each case. We see that this percentage is greater than 50% in all the cases. As before in section II C, in Supplementary Tables 2 and 3, we use the Wilcoxon signed-rank test. As can be seen from the Supplementary Table 2, the TN-GEO algorithm significantly outperforms the GTS, PBILD, and NADE-GEO methods on all performance metrics, rejecting the null hypothesis at the 0.05 significance level. On the other hand, the null hypotheses are accepted at $\alpha = 0.05$ for the TN-GEO algorithm over the other remaining algorithms. Thus, in terms of performance on all metrics combined, the results show that there is no significant difference between TN-GEO and these remaining seven SOTA optimizers (IPSO, IPSO-SA, GRASP, ABCFEIT, AAG, VNSQP, and ABC-HP).

Supplementary Table 3 shows the pairwise comparison of NADE-GEO against each of the SOTA optimizers and TN-GEO. The results show that NADE-GEO algorithm is aligned with the GTS, IPSO, IPSO-SA, and PBILD methods on all performance metrics retaining the null hypothesis at the 0.05 significance level. Thus, in terms of performance on all metrics combined, the results show that there is no significant difference between NADE-GEO and those optimizers. On the other hand, the null hypotheses are rejected at $\alpha = 0.05$ for the NADE-GEO algorithm over the other remaining algorithms, with NADE-GEO underperforming the remaining six SOTA optimizers (GRASP, ABCFEIT, AAG, VNSQP, and ABC-HP, and TN-GEO).

Supplementary Table 1. Detailed comparison with SOTA algorithms for each of the five index data sets and on seven different performance indicators described in Supplementary Note 1 B. Entries in *italic* correspond to cases where TN-GEO performed better or tied compared to the other algorithm. Entries in bold, corresponding to the best (lowest) value, for each specific indicator.

Data Set	Performance Indicator	GTS	IPSO	IPSO-SA	PBILD	GRASP	ABCFEIT	AAG	VNSQP	ABC-HP	NADE-GEO	TN-GEO
Hang Seng	Mean	1.0957	1.0953	-	*1.1431*	*1.0965*	1.0953	*1.0965*	*1.0964*	**1.0873**	*1.1007*	1.0958
	Median	*1.2181*	-	-	*1.2390*	1.2155	*1.2181*	*1.2181*	1.2155	**1.2154**	1.2170	1.2181
	Min	-	-	-	-	***0.0000***	***0.0000***	***0.0000***	***0.0000***	***0.0000***	***0.0000***	**0.0000**
	Max	-	-	-	-	***1.5538***	***1.5538***	***1.5538***	***1.5538***	***1.5538***	***1.5538***	**1.5538**
	MEUCD	-	-	***0.0001***	-	***0.0001***	***0.0001***	***0.0001***	***0.0001***	***0.0001***	***0.0001***	**0.0001**
	VRE	-	-	1.6368	-	*1.6400*	*1.6432*	*1.6395*	*1.6397*	**1.6342**	*1.6429*	1.6392
	MRE	-	-	0.6059	-	0.6060	0.6047	*0.6085*	0.6058	**0.5964**	0.6079	0.6082
DAX100	Mean	*2.5424*	*2.5417*	-	*2.4251*	2.3126	*2.3258*	2.3130	2.3125	**2.2898**	2.3125	2.3142
	Median	**2.5466**	-	-	*2.5866*	2.5630	*2.5678*	2.5587	2.5630	2.5629	2.5630	2.5660
	Minimum	-	-	-	-	*0.0059*	*0.0023*	*0.0023*	*0.0059*	*0.0059*	*0.0059*	**0.0023**
	Maximum	-	-	-	-	***4.0275***	***4.0275***	***4.0275***	***4.0275***	***4.0275***	***4.0275***	**4.0275**
	MEUCD	-	-	***0.0001***	-	***0.0001***	***0.0001***	***0.0001***	***0.0001***	***0.0001***	***0.0001***	**0.0001**
	VRE	-	-	*6.7806*	-	*6.7593*	*6.7925*	*6.7806*	*6.7583*	*6.8326*	*6.7591*	**6.7540**
	MRE	-	-	*1.2770*	-	*1.2769*	1.2761	*1.2780*	*1.2767*	**1.2357**	*1.2765*	1.2763
FTSE100	Mean	*1.1076*	*1.0628*	-	*0.9706*	*0.8451*	*0.8481*	*0.8451*	*0.8453*	**0.8406**	*0,8647*	0.8445
	Median	***1.0841***	-	-	***1.0841***	***1.0841***	***1.0841***	***1.0841***	***1.0841***	***1.0841***	***1.0841***	**1.0841**
	Minimum	-	-	-	-	0.0016	*0.0047*	**0.0006**	0.0045	0.0016	0.0016	0.0047
	Maximum	-	-	-	-	**2.0576**	2.0638	2.0605	2.0669	2.0670	*2.3718*	2.0775
	MEUCD	-	-	***0.0000***	-	***0.0000***	***0.0000***	***0.0000***	***0.0000***	***0.0000***	***0.0000***	**0.0000**
	VRE	-	-	*2.4701*	-	2.4350	*2.4397*	2.4350	2.4349	**2.4149**	*2.4713*	2.4342
	MRE	-	-	0.3247	-	0.3245	*0.3255*	**0.3186**	0.3252	0.3207	0.3235	0.3254
S&P100	Mean	*1.9328*	*1.6890*	-	*1.6386*	*1.2937*	*1.2930*	*1.2930*	**1.2649**	*1.3464*	*1.3314*	1.2918
	Median	*1.1823*	-	-	*1.1692*	1.1420	1.1369	**1.1323**	**1.1323**	*1.1515*	1.1420	1.1452
	Minimum	-	-	-	-	*0.0009*	***0.0000***	***0.0000***	***0.0000***	*0.0009*	*0.0009*	**0.0000**
	Maximum	-	-	-	-	*5.4551*	***5.4422***	*5.4642*	*5.4551*	*5.4520*	*6.7448*	5.4422
	MEUCD	-	-	***0.0001***	-	***0.0001***	***0.0001***	***0.0001***	***0.0001***	***0.0001***	***0.0001***	**0.0001**
	VRE	-	-	*2.6281*	-	2.5211	2.5260	2.5255	**2.5105**	*2.5364*	*2.5975*	2.5269
	MRE	-	-	0.7846	-	0.9063	0.8885	**0.7044**	0.9072	0.8858	0.9064	0.9117
Nikkei	Mean	*0.6732*	*0.6870*	-	*0.5972*	0.5782	0.5781	0.5781	*0.5904*	**0.5665**	*1.0391*	0.5793
	Median	*0.6066*	-	-	*0.5896*	*0.5857*	*0.5856*	0.5854	*0.5857*	*0.5858*	*0.6500*	0.5855
	Minimum	-	-	-	-	***0.0000***	***0.0000***	***0.0000***	***0.0000***	***0.0000***	*0.2270*	**0.0000**
	Maximum	-	-	-	-	***1.1606***	***1.1606***	*1.1607*	***1.1606***	***1.1606***	*7.4928*	**1.1606**
	MEUCD	-	-	***0.0000***	-	***0.0000***	***0.0000***	*0.0000*	***0.0000***	***0.0000***	***0.0000***	**0.0000**
	VRE	-	-	*0.9583*	-	*0.8359*	*0.8396*	**0.8191**	*0.8561*	0.8314	*1.6873*	0.8353
	MRE	-	-	*1.7090*	-	0.4184	0.4147	*0.4233*	0.4217	**0.4042**	*0.4256*	0.4229

Supplementary Table 2. Pairwise comparison of TN-GEO against each of the SOTA optimizers. The asymptotic significance is part of the Wilcoxon signed-rank test results. The null hypothesis that the performance of the two algorithms is the same is tested at the 95% confidence level (significance level: $\alpha = .05$). Results show that TN-GEO is on par with all the SOTA algorithms, and in two cases, GTS and PBILD, it significantly outperforms them. We also report the count for TN-GEO wins, losses, and ties, compared to each of the other algorithms.

TN-GEO vs Other:	GTS	IPSO	IPSO-SA	PBILD	GRASP	ABCFEIT	AAG	VNSQP	ABC-HP	NADE-GEO
Wins(+)	6	4	6	9	12	10	11	11	8	8
Loss(-)	2	1	4	0	12	9	11	12	16	18
Ties	2	0	5	1	11	16	13	12	11	9
(Wins+Ties)/Total	80%	80%	67%	100%	66%	74%	69%	66%	54%	77%
Asymptotic significance (p)	.036	.080	.308	.008	.247	.888	.363	.594	.110	.003
Decision	Reject	Retain	Retain	Reject	Retain	Retain	Retain	Retain	Retain	Reject

Supplementary Table 3. Pairwise comparison of NADE-GEO against each of the SOTA optimizers and TN-GEO. The asymptotic significance is part of the Wilcoxon signed-rank test results. The null hypothesis that the performance of the two algorithms is the same is tested at the 95% confidence level (significance level: $\alpha = .05$). Results show that NADE-GEO is on par with the first four, but underperforming the remaining six SOTA optimizers.

NADE-GEO vs Other:	GTS	IPSO	IPSO-SA	PBILD	GRASP	ABCFEIT	AAG	VNSQP	ABC-HP	TN-GEO
Wins(+)	5	3	4	7	4	7	5	4	3	8
Loss(-)	4	2	4	2	17	19	21	19	20	18
Ties	1	0	4	1	14	9	9	12	12	9
(Wins+Ties)/Total	60%	60%	67%	80%	51%	46%	40%	46%	43%	49%
Asymptotic significance (p)	.594	.500	.919	.314	$< .001$	0.002	$< .001$	$< .001$	.001	.003
Decision	Retain	Retain	Retain	Retain	Reject	Reject	Reject	Reject	Reject	Reject

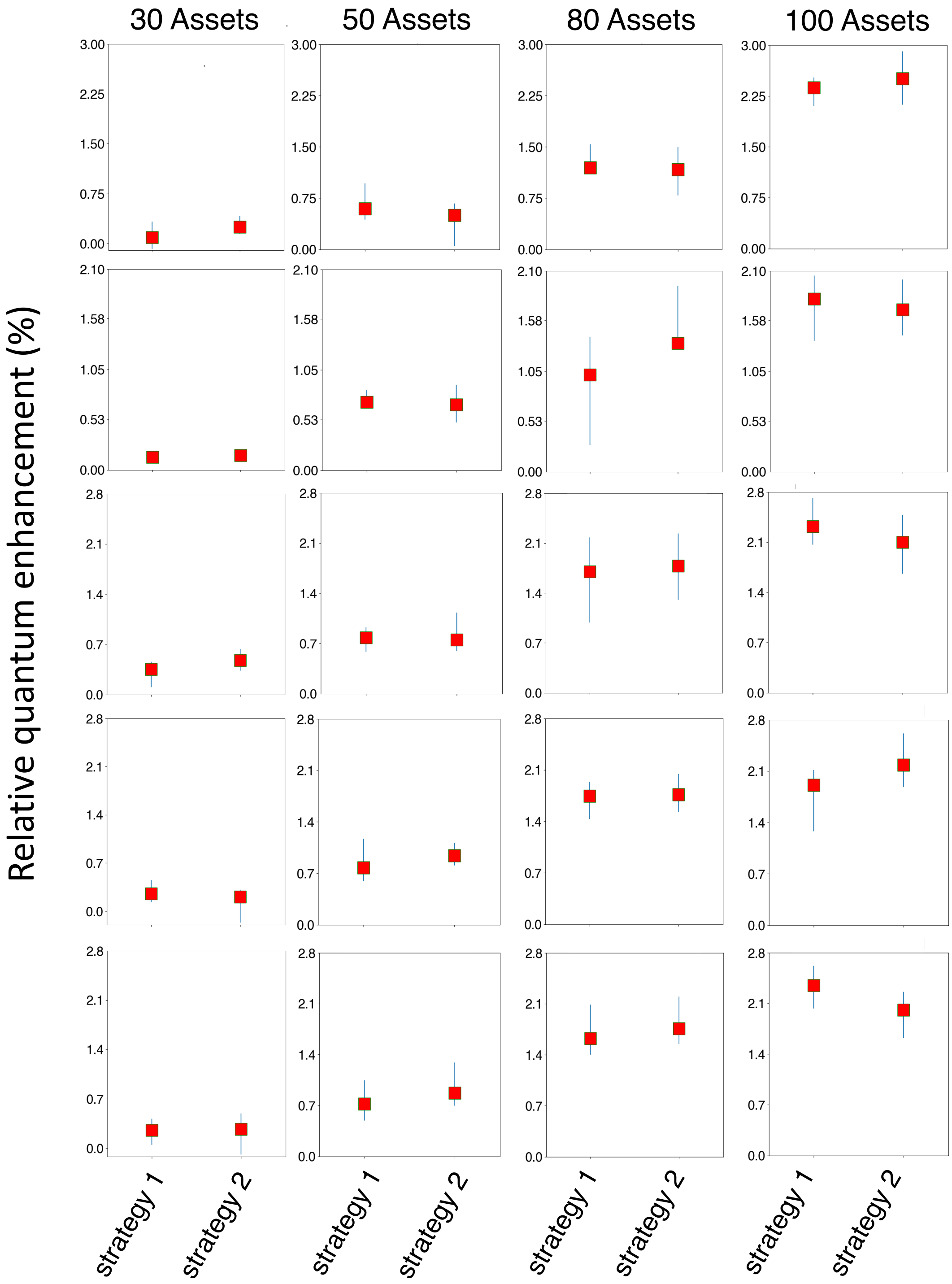

Supplementary Figure 2. Relative TN-GEO enhancement similar to those shown in the bottom panel of Fig. 2 in the main text. For these experiments, portfolio optimization instances with a number of variables ranging from $N = 30$ to $N = 100$ were used. Here, each panel corresponds to a different investment universes corresponding to a random subset of the S&P 500 market index. Note the trend for a larger quantum-inspired enhancement as the number of variables (assets) becomes larger, with the largest enhancement obtained in the case on instances with all the assets from the S&P 500 ($N = 500$), as shown in Fig. 2 in the main text.

[1] Joachim Dahl Martin Andersen and Lieven Vandenberghe, "Python software for convex optimization," http://cvxopt.org (2020).
[2] T-J Chang, Nigel Meade, John E Beasley, and Yazid M Sharaiha, "Heuristics for cardinality constrained portfolio optimisation," Computers & Operations Research **27**, 1271–1302 (2000).
[3] Tunchan Cura, "Particle swarm optimization approach to portfolio optimization," Nonlinear analysis: Real world applications **10**, 2396–2406 (2009).
[4] The GPyOpt authors, "Gpyopt: A bayesian optimization framework in python," http://github.com/SheffieldML/GPyOpt (2016).
[5] Zhao-Yu Han, Jun Wang, Heng Fan, Lei Wang, and Pan Zhang, "Unsupervised generative modeling using matrix product states," PRX **8**, 031012 (2018).
[6] Marcello Benedetti, Delfina Garcia-Pintos, Oscar Perdomo, Vicente Leyton-Ortega, Yunseong Nam, and Alejandro Perdomo-Ortiz, "A generative modeling approach for benchmarking and training shallow quantum circuits," npj Quantum Information **5**, 45 (2019).
[7] Ignacio Cirac, David Perez-Garcia, Norbert Schuch, and Frank Verstraete, "Matrix product states and projected entangled pair states: Concepts, symmetries, and theorems," (2020), arXiv:2011.12127 [quant-ph].
[8] "Code for unsupervised generative modeling using matrix product states," https://github.com/congzlwag/UnsupGenModbyMPS (2018).
[9] Benigno Uria, Marc-Alexandre Côté, Karol Gregor, Iain Murray, and Hugo Larochelle, "Neural Autoregressive Distribution Estimation," (2016), arXiv:1605.02226.
[10] Matthew T. Perry and Richard J. Wagner, "Python module for simulated annealing," https://github.com/perrygeo/simanneal (2019).
[11] Javier Alcazar, Vicente Leyton-Ortega, and Alejandro Perdomo-Ortiz, "Classical versus quantum models in machine learning: insights from a finance application," Machine Learning: Science and Technology **1**, 035003 (2020).
[12] Guang-Feng Deng, Woo-Tsong Lin, and Chih-Chung Lo, "Markowitz-based portfolio selection with cardinality constraints using improved particle swarm optimization," Expert Systems with Applications **39**, 4558–4566 (2012).
[13] M Mozafari, F Jolai, and S Tafazzoli, "A new ipso-sa approach for cardinality constrained portfolio optimization," International Journal of Industrial Engineering Computations **2**, 249–262 (2011).
[14] Khin Lwin and Rong Qu, "A hybrid algorithm for constrained portfolio selection problems," Applied intelligence **39**, 251–266 (2013).
[15] Adil Baykasoğlu, Mualla Gonca Yunusoglu, and F Burcin Özsoydan, "A grasp based solution approach to solve cardinality constrained portfolio optimization problems," Computers & Industrial Engineering **90**, 339–351 (2015).
[16] Can B Kalayci, Okkes Ertenlice, Hasan Akyer, and Hakan Aygoren, "An artificial bee colony algorithm with feasibility enforcement and infeasibility toleration procedures for cardinality constrained portfolio optimization," Expert Systems with Applications **85**, 61–75 (2017).
[17] Can B Kalayci, Olcay Polat, and Mehmet A Akbay, "An efficient hybrid metaheuristic algorithm for cardinality constrained portfolio optimization," Swarm and Evolutionary Computation **54**, 100662 (2020).
[18] Mehmet Anil Akbay, Can B Kalayci, and Olcay Polat, "A parallel variable neighborhood search algorithm with quadratic programming for cardinality constrained portfolio optimization," Knowledge-Based Systems **198**, 105944 (2020).
[19] Tunchan Cura, "A rapidly converging artificial bee colony algorithm for portfolio optimization," Knowledge-Based Systems **233**, 107505 (2021).
